# Treatment allocation strategies for umbrella trials in the presence of multiple biomarkers: A comparison of methods

**DOI:** 10.1002/pst.2119

**Published:** 2021-03-24

**Authors:** Luke Ondijo Ouma, Michael J. Grayling, Haiyan Zheng, James Wason

**Affiliations:** 1 Biostatistics Research Group, Population Health Sciences Institute, Newcastle University, Newcastle upon Tyne, UK; 2 MRC Biostatistics Unit, University of Cambridge, Cambridge, UK

**Keywords:** adaptive design, adaptive randomisation, constrained randomisation, patient allocation, precision medicine, stratified randomisation

## Abstract

Umbrella trials are an innovative trial design where different treatments are matched with subtypes of a disease, with the matching typically based on a set of biomarkers. Consequently, when patients can be positive for more than one biomarker, they may be eligible for multiple treatment arms. In practice, different approaches could be applied to allocate patients who are positive for multiple biomarkers to treatments. However, to date there has been little exploration of how these approaches compare statistically. We conduct a simulation study to compare five approaches to handling treatment allocation in the presence of multiple biomarkers – equal randomisation; randomisation with fixed probability of allocation to control; Bayesian adaptive randomisation (BAR); constrained randomisation; and hierarchy of biomarkers. We evaluate these approaches under different scenarios in the context of a hypothetical phase II biomarker-guided umbrella trial. We define the pairings representing the pre-trial expectations on efficacy as linked pairs, and the other biomarker-treatment pairings as unlinked. The hierarchy and BAR approaches have the highest power to detect a treatment-biomarker linked interaction. However, the hierarchy procedure performs poorly if the pre-specified treatment-biomarker pairings are incorrect. The BAR method allocates a higher proportion of patients who are positive for multiple biomarkers to promising treatments when an unlinked interaction is present. In most scenarios, the constrained randomisation approach best balances allocation to all treatment arms. Pre-specification of an approach to deal with treatment allocation in the presence of multiple biomarkers is important, especially when overlapping subgroups are likely.

## 1 Introduction

Recent years have seen an increasing interest in the development of targeted therapies that may provide substantial clinical benefit in specific patient subgroups (e.g., higher response rates, longer progression free survival, or improved overall survival). Consequently, the early phases of drug development are now more commonly being designed to efficiently learn about the utility of experimental therapies for specific patient subgroups. In such precision medicine trials, the principal idea is to tailor treatment strategies according to individual patient characteristics; often predictive biomarkers.^1,2^ In oncology, for example, epidermal growth factor receptor (EGFR) and ALK alterations as well as BRAF and HER2 mutations are known biomarkers, used to guide the development of targeted treatments in recent and ongoing cancer trials.^3–5^ These precision medicine trials are commonly implemented using an innovative single trial infrastructure and protocol,^6^ where multiple sub-studies in a single trial can: (a) evaluate multiple treatments within a single disease context (so-called umbrella and platform trials); or (b) evaluate a treatment simultaneously in different patient subgroups (so-called basket trials).

Our focus here will be on one of these novel approaches: the umbrella trial design. Umbrella trials, as noted above, are designed to evaluate multiple interventions (single agent or drug combinations) matched with the subtypes of a single disease population.^6,7^ For example, in the ALCHEMIST umbrella trial of NSCLC patients, two candidate biomarkers define the sub-studies: (a) anaplastic lymphoma kinase (ALK) translocations and (b) EGFR. Only patients positive for one of the targeted biomarkers received the biomarker linked treatments; crizotinib and erlotinib.^3^ To date, most umbrella trials have been implemented to investigate cancer therapies,^8^ owing to significant progress in the identification of tumour genomic drivers and the development of molecularly targeted agents. Nonetheless, they have utility outside of oncology in settings like rheumatology where subtypes of a chronic disease can be targeted by biomarker-guided treatments.^9^

However, one potential concern in the design of umbrella trials is that patients may test positive for more than one biomarker that guides which targeted therapy should be received. For instance, in attention deficit hyperactivity disorder and autism spectrum disorder, multiple biomarkers have been identified with potential to guide treatment, most of which co-occur within the same patient.^10^ Furthermore, in oncology umbrella trials, multiple tumour mutations can co-occur; meaning patients could test positive for more than one biomarker that defines the treatment arms. In line with standard enrolment criteria to an umbrella trial, such patients are eligible for multiple sub-studies, hence treatment allocation for such individuals is not obvious.

In recent umbrella trials, different approaches have been taken to allocating treatments to patients with multiple biomarkers. In the Lung-MAP trial, patients eligible for more than one subgroup have higher chance to be randomised between eligible sub-studies with lower biomarker prevalence (i.e., probability of assignment to a specific sub-study is inversely proportional to biomarker prevalence).^11^ Precisely, the frequency of overlaps between biomarkers is updated during the trial for the different biomarkers and a predefined algorithm facilitates enrolment of such patients to trial arms.^12^ In the FOCUS-4 trial, the molecular cohorts (BRAF, *PIK3CA or PTEN loss, KRAS or NRAS* and all wild type) are arranged in a hierarchy, such that for example a patient with both a *BRAF* mutation and a *KRAS* mutation will always be classified into the *BRAF* mutation cohort.^13^ Finally, should multiple targeted molecular aberrations be present in the recently proposed PANGEA trial, a 9-point pre-specified prioritisation algorithm will be used to allocate these patients, with priority given to higher allele frequency (for mutations) or higher gene copy/expression.^4^ Finally, the ISPY2 trial^14^ uses a Bayesian adaptive randomisation (BAR) procedure to assign patients to treatments. This approach naturally deals with the problem of multiple biomarkers as patients are assigned to treatments for which they are most likely to respond.

However, it is noteworthy that in several ongoing or recently published umbrella trials, there is limited information on how patients would be allocated to treatments when they are positive for multiple biomarkers. This may be attributable to: (a) the fact that no patients may have tested positive thus far for multiple biomarkers (see, e.g., the ALCHEMIST trial^3^); (b) there is no knowledge of the best approach to guide the chosen allocation rule; or (c) such treatment decisions are left to the trial physician/clinician.

In this work, we seek to address point (2); the fact that it is currently unclear how the method used to account for multiple biomarkers will affect the statistical properties of the trial. Specifically, in exploring this issue we hope to investigate whether there is a method that generally works well in most plausible scenarios. To do this, we conduct a simulation study to compare five different possible approaches that have been or could be implemented to guide the allocation of patients to treatments in the presence of multiple biomarkers in an umbrella trial. We evaluate the operational characteristics of each of these approaches and their respective limitations.

## 2 Methods

### 2.1 Trial design and allocation strategies

**Figure 1:**
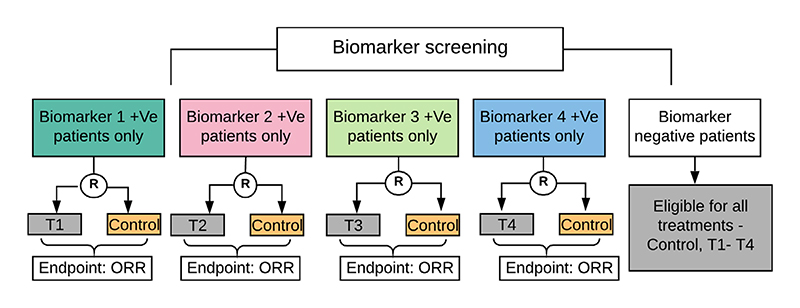
Illustrative schema of the motivating umbrella design, wherein several treatment allocation strategies are assessed

An illustration of the motivating umbrella design for this simulation study is presented in Figure 1. For this paper we restrict our focus to a design setting in which there are four biomarkers (*B*_1_, *B*_2_, *B*_3_, *B*_4_), four experimental treatments (*T*_1_, *T*_2_, *T*_3_ and *T*_4_), and a single control (*T*_0_). Each experimental treatment is linked to a specific biomarker. The pairings representing the pre-trial expectations on efficacy are referred to as *linked pairs*, and we suppose that treatment *T_i_* and biomarker *B_i_* are a linked pair for *i* = 1, …, 4. We refer to the other biomarker-treatment pairings as *unlinked*.

At the start of the trial, patients are screened for each of the four biomarkers. Patients who test positive for only one of the biomarkers are eligible for control and the biomarker-linked treatment. For patients with multiple biomarkers of interest, one of the different treatment allocation strategies explained in detail below is used to allocate them to a treatment arm.

We suppose a maximum of 400 patients are to be recruited over the trial′s duration. The primary endpoint in each sub-study is assumed to be common and binary, to reflect the typical use of objective tumour response by RECIST in phase II oncology trials.^15^

We stipulate that patients who are negative for all the targeted biomarkers are eligible for all experimental treatments (plus control), as in a recently proposed adaptive design for biomarker trials with linked treatments.^16^ Note that this is in contrast to the Lung-MAP trial where patients with no targeted biomarker are assigned to a ‘no-match’ sub-group.^11^ It is further assumed that the prevalence of the biomarkers is well understood, herein assumed to be 0.3 for *B*_1_ and *B*_3_ and 0.25 for *B*_2_ and *B*_4_, with positivity for *B_i_* independent of positivity for *B_j_*, *j* ≠ *i*.

We note that this motivating trial design is a subset of pragmatic umbrella designs that could be used in practice, which could either comprise single-arm sub-trials or even where biomarker negative patients comprise their own non-match subgroup.

In the simulation study, we evaluate five different approaches to treatment allocation for the patients who test positive for multiple biomarkers. Patients who test positive for one biomarker are allocated to treatment in the same way across all five methods. Specifically, they are equally randomised between the two treatments they are eligible for: the biomarker-linked treatment and control.

#### 2.1.1 Equal randomisation

In this approach, patients who are positive for multiple biomarkers, say *B_i_* and *B_j_* (*j* ≠ *i*), have equal probability to be randomised between all experimental treatments for which they are eligible, and the control treatment (i.e., *T_i_*, *T_j_*, and *T*_0_). This is an easy-to-use approach when the prevalence of patients with multiple biomarkers is fairly low and when the relative importance of biomarkers is relatively unknown.

#### 2.1.2 Randomisation with fixed probability of allocation to control

This is a slightly different version of the equal randomisation procedure. For patients positive for multiple biomarkers, the probability of allocation to the control is fixed at *θ* (0 < *θ* < 1), and the rest of the probability is equally split between experimental treatments for which they are eligible. The operational characteristics of this approach are assessed for different values of the fixed probability *θ*.

#### 2.1.3 Hierarchy of biomarkers

In this case, biomarkers are ordered by their relative importance. Without loss of generality, we assume that the hierarchy is *B*_1_, *B*_2_, *B*_3_ and *B*_4_ where *B*_1_ is anticipated to be the most predictive of treatment response. If a patient is positive for two biomarkers, say *B_i_* and *B_j_* (*i* < *j*), with a probability *ρ* (0.5 ≤ *ρ* ≤ 1) such patients would be randomised equally between *T_i_* and *T*_0_, and with probability 1 – *ρ* they are randomised equally between *T_j_* and *T*_0_. If a patient is positive for more than two biomarkers, say *B_i_*, *B_j_*, and *B_k_* (*i* < *j* < *k*), with probability *ρ* they are randomised equally between *T_i_* and *T*_0_, and with probability 1 – *ρ* they are randomised equally between *T_j_*, *T_k_*, and *T*_0_. A similar statement holds for those patients positive for all four biomarkers. Here, the choice of *ρ* indicates the level of confidence in the pre-specified hierarchy. The special case when *ρ* = 1 corresponds to complete belief; this has been used in practice in the FOCUS-4 trial. A hierarchy approach may be particularly useful when the hierarchy reliably reflects the predictability of such biomarkers. However, this procedure can only be employed when a plausible hierarchy of biomarkers has been set up by the trial investigators.

#### 2.1.4 Constrained randomisation

This approach prioritises the allocation to a treatment arm with a lower patient accrual (with probability 0.5 ≤ *ϕ* ≤ 1), when patients are positive for more than one biomarker and thus eligible for multiple biomarker-linked treatments. That is, if a patient is positive for two biomarkers, say *B_i_* and *B_j_* (*j* ≠ *i*), and *n_i_* and *n_j_* patients have been allocated thus far to the respective treatment arms *T_i_* and *T_j_*, then with probability ϕ such a patient would be randomised equally between control and treatment *T_i_* (*T_j_*) for *n_i_* < *n_j_* (*n_j_* < *n_i_*). When *ϕ* = 1, if *n_i_* and *n_j_* patients have been allocated to treatment subgroups *T_i_* and *T_j_* respectively, a patient positive for biomarkers *T_i_* and *T_j_* is always allocated to subgroup *T_j_* if *n_j_* < *n_i_* at that allocation time point. This approach is likely to be advantageous in scenarios when accrual to certain arms may be low during the trial.

#### 2.1.5 Bayesian adaptive randomisation

BAR assigns patients to the most promising treatment (or combination of treatments) on the basis of accumulating data as the trial proceeds. We base our considered BAR procedure on that previously proposed by Wason et al^16^ modifying this method in two ways: (a) in the first stage, our method initiates with the equal randomisation procedure described above; (b) allocation probabilities are updated at interim analyses to guide treatment allocation in later stages for patients with multiple biomarkers only. Patients with a single biomarker are eligible only for the linked experimental treatment or control throughout the trial.

The BAR procedure is embedded with four interim analyses, to allow adaptations that utilise accruing information to allocate patients with multiple biomarkers to appropriate treatment arms. We allow no interim analysis for the other four approaches as those do not involve any adaptation. More precisely, the BAR design is implemented with interim analyses after 100, 175, 250 and 325 patients have been recruited, as previous work has shown that there is diminishing returns for a higher number of interim analysis.^17^ At each interim analysis, a Bayesian equivalent of the logistic regression model below is fitted to estimate the probability of response. The final analysis is still performed in a frequentist framework.

For all of the parameters of the Bayesian model, we specify uninformative uniform prior distributions *U*(−10, 10). While it is theoretically possible to use informative priors for linked biomarker-treatment interaction parameters, we do not focus on this here so as to provide a more broadly applicable perspective.

### 2.2 Hypothesis testing

Denoting the treatment response of patient *i* by *Y_i_*, we assume *Y_i_* ~*Bern*(*p_i_*). A logistic regression model is fitted to evaluate *p_i_*, the probability of response, as a function of the treatment effects, biomarker effects, and interactions between biomarkers and treatments:

(1) $$\text{log}\left(\frac{p_{i}}{1-p_{i}}\right)=\alpha +\sum_{k=1}^{K}{\text{T}}_{ik}\beta_{k}+\sum_{l=1}^{K}\chi_{il}\gamma_{l}+\sum_{k=1}^{K}\sum_{l=1}^{K}{\text{T}}_{ik}\chi_{il}\delta_{kl}.$$

Here *K* = 4 is the number of experimental treatments; *α* is an intercept term (i.e., the log-odds of treatment response for a patient positive for no biomarkers allocated to control); *T_ik_* takes the value 0 or 1 representing whether patient i is allocated to treatment *k*; *β_k_* represents the main effect of treatment *k*; *x_il_* takes the value 0 or 1 representing whether patient i is positive for biomarker *l*; *γ_l_* represents the main effect of biomarker *l*; and *δ_kl_* is the interaction effect between treatment *k* and biomarker *l*.

A total of *K*(*K* + 1) = 20 hypotheses are to be tested; of interest is whether an experimental treatment provides benefit over control among patients positive for a given biomarker. Thus, we perform a one-sided test (at 5% significance level) of the null hypothesis that experimental treatment *k* provides no benefit over control in patients positive for biomarker *l* for all *k* and *l*:

$$H_{0}^{\left(k,l\right)}:\{\begin{array}{ll} \beta_{k}+\delta_{kl}\leq 0 & \text{if}\;l>0,\\ \beta_{k}\leq 0 & \text{if}\;l=0. \end{array}$$

For identifiability of the model we specify *δ_kk_* = 0.

The probability of treatment *k* being identified as better than control in biomarker population l is referred to in general as ‘statistical power’ (i.e., the probability $H_{0}^{\left(k,l\right)}$ is rejected is the statistical power for treatment k in biomarker group l). When we refer to a type I error, this corresponds to the probability of a treatment being determined to be better than control in a particular biomarker population when in fact this is not the case (i.e., the probability a particular $H_{0}^{\left(k,l\right)}$ is rejected when this hypothesis is true). We do not consider the family-wise error-rate.

The code to implement this simulation study (using R version 3.6.3 and JAGS) is available on GitHub (https://github.com/oondijo/multipleBiomarkers).

## 3 Results

We simulated 10,000 virtual trials for each of the five treatment allocation approaches under 12 different simulation scenarios (see Supporting Information). As shown in Table S1, these simulation scenarios are for various configurations of values for *β_k_* and *δ_kl_*, that is, the treatments being (in)effective and whether there exists a treatment-biomarker interaction. More specifically, they include cases where the biomarker-linked treatments work as expected, and where the anticipated biomarker-treatment interactions are incorrect but instead an unanticipated unlinked treatment works. The true parameter values were chosen to guarantee at least 80% power for all the treatment allocation approaches when a true treatment-biomarker interaction exists.

For the hierarchy, constrained randomisation, and randomisation with fixed allocation probability to control approaches, we additionally examined the operational characteristics by varying the allocation probabilities *ρ*, *ϕ* and *θ*; specifically, we considered *ρ* = 0.5,0.75,0.9, *ϕ* = 0.5,0.75,0.9, and *θ* = 0.2,0.25,0.3. The simulation study evaluated various operating characteristics of the different approaches, including: statistical power and the average number and proportion of (a) patients on an experimental treatment; (b) patients on the best treatment available to them; (c) patient responses; (d) patients on each treatment; and (e) bias and mean squared error (MSE).

### 3.1 Patients on treatment

Table 1 shows the proportion of patients on an experimental treatment (i.e., not on the control) on average. The constrained randomisation approach allocates the highest number of patients to experimental treatments (at least 80%). This is unsurprising as this procedure mirrors the minimization approach where at any stage of the trial, patients are preferentially allocated to treatment arms that have accrued fewer number of patients. Only the approach of randomisation with fixed probability of allocation to the control (when *θ* = 0.2) posts similar results. Overall, the hierarchy approach allocates the smallest proportion of patients to experimental treatment on average, although this increases as the degree of belief on the validity of the hierarchy increases. This is reasonable: in a hierarchy approach, patients are increasingly randomised between the control and the biomarker-linked treatment that is most important (or most predictive of a good response) from the pool of biomarker-linked treatments that they are eligible for.

### 3.2 Patients on the best treatment available to them

In Table 2, we report the proportion of all patients on the best treatment available to them, whenever there is a biomarker-linked treatment superior to the rest; here, ‘superior’ means the treatment induces a higher *p_i_*. In scenario 1, all experimental treatments have the same effect as the control, hence none of the treatments is considered superior. In such a case, patients receiving an experimental treatment, or the control would have equal probability of a good outcome. In scenario 2, T_1_ induces the highest probability of response for biomarker profiles P9-P14, hence the best treatment among the pool of treatments they are eligible for (Figure S1).

For the BAR treatment allocation strategy, we evaluated this characteristic in a slightly different fashion, assessing the number of patients on the best treatment on average at each stage of the trial. We illustrate graphically in the Figure S1 what is meant by the best treatment for each biomarker profile and across all simulation scenarios.

When there is an interaction between a linked pair (scenario 2), the randomisation approach with fixed allocation probability to control (*θ* = 0.2) allocates the highest proportion of patients to the best treatment available to them. In other scenarios, such as when there is an interaction between an unlinked pair (scenario 3), all approaches perform poorly in assigning patients to the best treatment (approximately 2%).

The BAR method outperforms the alternative approaches whenever a linked treatment has a detrimental effect on patients, for whom a treatment benefit was anticipated. In scenario 4, when *T*_1_ has a detrimental effect on biomarker 1 positive patients, at least 90% of the patients are allocated to the best treatment that is available for them. This is because it makes use of accruing trial data to allocate patients to treatments that are better for them as the trial progresses.

### 3.3 Statistical power

Table 3 provides a summary of how the statistical power varies across these treatment allocation approaches. When there is a positive interaction between a biomarker and its linked treatment (in scenarios 2, 8 and 12), the BAR and hierarchy approaches have the highest statistical power compared with the alternative allocation strategies. The allocation strategies of equal randomisation and randomisation with fixed allocation probability to the control have low absolute differences in power (between 2 and 4%) relative to the best two approaches in detecting a linked interaction. For the hierarchy procedure, this tends to be particularly advantageous when the specified hierarchy correctly reflects the validity of biomarkers. This is evidenced by results in scenarios 2, 8 and 12 where patients have high chance of being allocated to the most promising biomarker-linked treatment among all available ones (*ρ* = 0.9). When the probability of the specified hierarchy being correct reduces to 50%, we observe losses in power (around 3–5%) for the three scenarios above (see Table S6).

In scenario 3, when there is an unanticipated interaction between *T*_1_ and *B*_2_, the hierarchy approach still performs better than the alternative approaches. This is because patients who test positive for both *B*_1_ and *B*_2_ would be allocated to *T*_1_ following the specified hierarchy. Nevertheless, we observe a considerable decrease in power, compared to scenarios with a linked pair where the specified hierarchy is plausible. By contrast, when it is the other way around and *T*_2_ works in *B*_1_ patients (scenario 10), the hierarchy procedure leads to the least satisfactory performance. In such a case, the constrained randomisation procedure seems to work better, although comparatively it has the lowest power for detecting a linked interaction (at least 72%, with differences in power of between 7 and 10% compared to the BAR and hierarchy procedures).

**Figure 2:**
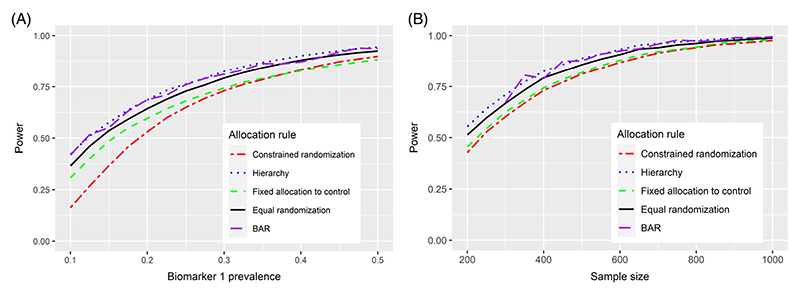
Statistical power of the five-treatment allocation approaches as (A) biomarker prevalence and (B) sample size varies under scenario 2

We further examined how the statistical power of the five treatment allocation strategies to recommend *T*_1_ in *B*_1_ positive patients (scenario 2) varies as: (a) the prevalence of *B*_1_ varies between 0.1 and 0.5 and (b) the overall sample size changes from 200 to 1000 in increments of 50. The results presented in Figure 2A, B indicate that the statistical power of all the five approaches strongly relies on both the sample size and the biomarker prevalence. All the treatment allocation strategies have less than 50% power when the prevalence of *B*_1_ is 0.1, but this increases to 80–90% as the prevalence approaches 0.5. Notably, the relative performance of each method to one another remains constant as we vary prevalence and sample size.

### 3.4 Patient response and treatment allocation

We provide in the Supporting Information results showing the overall patient allocation and patient response in each of the different treatment arms. The results show that the constrained randomisation procedure allocates on average a higher proportion of patients to experimental treatments. This is because this approach mirrors a minimization procedure where treatment arms that have accrued fewer patients are prioritised in the event a patient is eligible for multiple treatments.

### 3.5 Bias and mean square error

**Figure 3:**
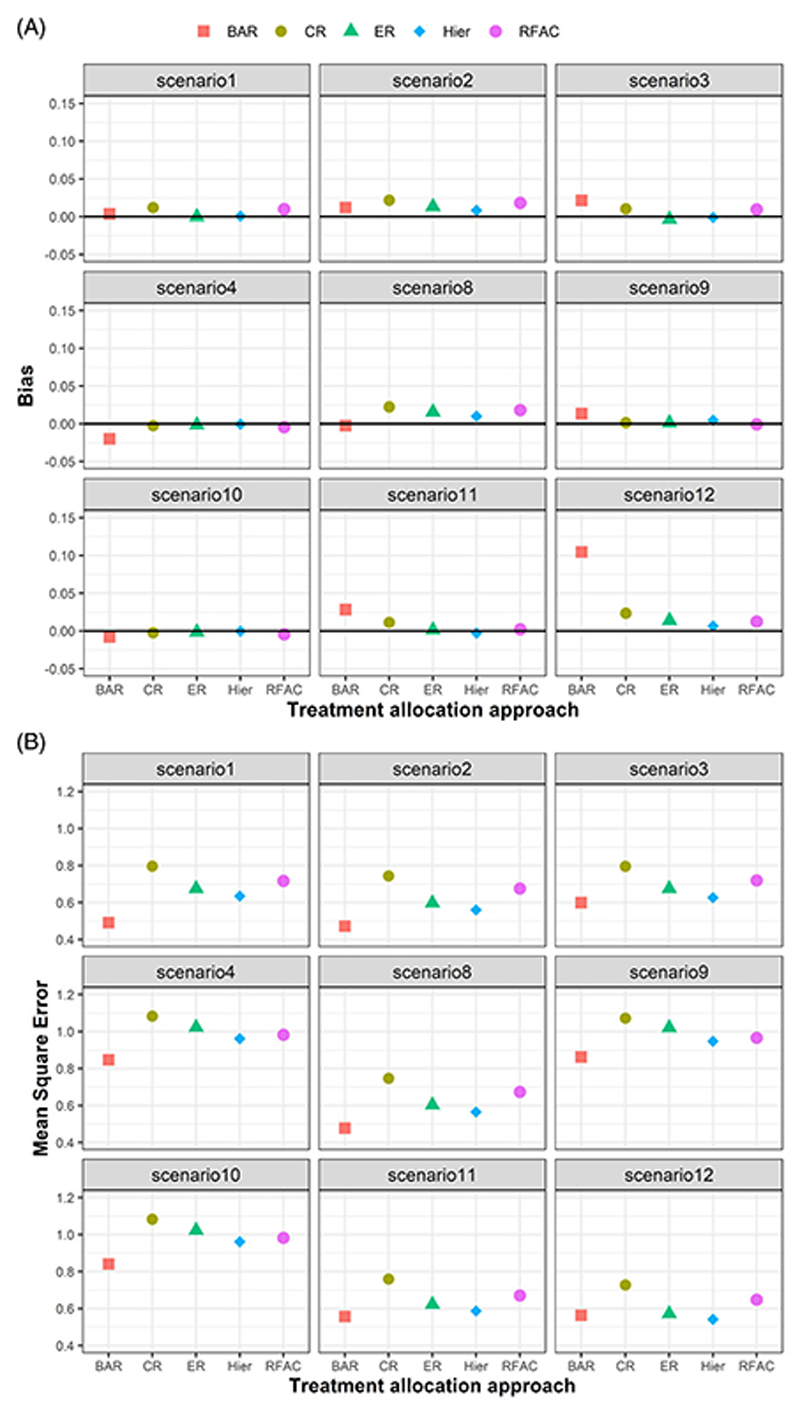
Bias and mean squared error of the point estimate for δ11 based on the five treatment allocation approaches

We additionally compare the five treatment allocation approaches in terms of bias and MSE of their point estimators (posterior means) for the treatment effects. Figure 3 shows all approaches produce reasonable bias in all scenarios. The BAR approach produces larger bias and the smallest MSE across most scenarios. On the other hand, point estimators based on CR have slightly higher variability (and MSE in scenarios 1, 2 and 8) compared to Hier, ER, RFAC and BAR.

## 4 Discussion

Umbrella trials offer the promise of answering multiple treatment-related questions within a single disease setting, but are accosted by challenges that play an important role in their design. In this paper, we have compared five different approaches to allocate treatments to patients in an umbrella trial in the presence of multiple biomarkers. We have considered a trial design where the prevalence of each biomarker, and the overlaps, is known at the design stage, and that prior to trial initiation there is substantial evidence for experimental treatment benefit in biomarker linked subgroups.

Our simulation results show that the approaches of pre-specifying the hierarchy of biomarkers and BAR have the highest power to detect a linked interaction. The performance of the hierarchy approach is however dependent on the degree of belief on the validity of the pre-specified hierarchy of biomarkers. In practice, a hierarchy approach such as the one previously suggested in the FOCUS4 trial^18^ assumes that the hierarchy of biomarkers is entirely correct, a special case of what we have evaluated in our study. We note, however, that there may be a degree of uncertainty in this pre-specified hierarchy. This uncertainty may increase when the hierarchy consists of several biomarkers and/or there is insufficient evidence to quantify the level of importance of a given set of biomarkers. The choice of the degree of belief on the hierarchy is information that could be elicited from experts prior to the design of a trial.

Although the BAR approach has been used in recent trials (e.g., in the recent BATTLE trials^19^), it has not been explicitly used to guide treatment allocation for patients with multiple biomarkers only. The approach we use here is slightly different from a previously proposed linked-BAR approach^16^ where BAR was used to guide treatment allocation for all patients. Unlike the hierarchy approach, the BAR method is more robust to the biomarker ordering being invalid. As expected, we note that the BAR approach yields comparatively larger bias in point estimators than non-adaptive randomisation approaches as shown in previous simulation studies.^20,21^ Our simulation results show that BAR is a particularly beneficial approach when a treatment delivers an unanticipated detrimental effect, as it allows mid-trial modifications to enable more patients with multiple biomarkers to be allocated to promising treatments. Its limitation is that setting up a BAR approach requires prior knowledge about how this procedure works and specification of plausible parameter estimates.

The approaches of equal randomisation and randomisation with fixed allocation probability to the control are conceptually similar. They both have comparable power to BAR except when there is an interaction between an unlinked pair. When recruitment to certain treatment(s) is low, the constrained randomisation approach shows the most promising results in balancing allocation to treatment arms. However, if low accrual is attributed to a lower than anticipated biomarker prevalence, this approach should be considered carefully.

Ultimately, none of the approaches we have considered is optimal in all settings. The choice of an approach may considerably influence the operational characteristics of a trial and should be considered carefully. For instance, the equal randomisation approach maintains reasonable statistical properties in most scenarios and is easy to use in practice, but when a substantial proportion of patients test positive for multiple biomarkers, it may not be the most reliable approach. In such cases, the hierarchy or BAR approaches often perform better, although for the former the biomarker hierarchy should be supported by reliable clinical evidence.

In practice, there may be uncertainty in the estimate of true prevalence of the overlaps when there are several biomarker-defined subgroups. This may warrant the use of an approach that accommodates use of accruing trial data to update the prevalence of overlaps, such as in the LUNG-MAP umbrella trial.^11^ We further acknowledge that there could be variations to our motivating trial design. For example, biomarker negative patients may comprise either a non-match subgroup or could be entirely excluded from the trial. In certain disease settings such as oncology, the control treatment may even be different in the various subgroups.

Our findings are, however, readily applicable to settings where umbrella trial arms are not necessarily biomarker-defined, though the subgroup defining characteristic defines which treatment a patient receives. An example could be an umbrella trial in the neurological disorders setting, several of which such disorders can co-occur within the same patient and share common symptoms. Although we have only focused on binary biomarkers, our results may also be relevant in the context of continuous markers, which are often handled through dichotomisation.

Our comparison of methods is also constrained by non-statistical issues. Multiple biomarkers can occur at a high and low frequency respectively. In other contexts, two biomarkers may be mutually exclusive, or a particular biomarker may be present if and only if a certain other biomarker is present. Another potential concern is intra-patient heterogeneity, where a patient's biomarker profile may change over the trial duration. In such cases, some trials like the PANGEA-BBP trial^4^ propose sequential biopsy and treatment reassignment, which ultimately may help deal with the problem of multiple biomarkers.

We conclude that in the design of an umbrella trial, when patients are likely to test positive for multiple biomarkers, it is important to pre-specify an approach that allows optimal treatment allocation. This needs to be considered in the context of the trial sample size, prevalence of the biomarkers, and prevalence of individual overlaps within the patient population.

## Supplementary Material

Supplementary File

## Figures and Tables

**Table 1:** Average proportion of patients on experimental treatment

Equal randomisation		Proportion (range) 63.9% (52.7–72.5)
Randomisation with fixed allocation probability to control	*θ* = 0.2	80.0% (71.2–87.5)
	*θ* = 0.25	75.0% (66–83)
	*θ* = 0.3	70.0% (60.5–78.8)
Hierarchy	*ρ* = 0.5	64.5% (54.8–72.7)
	*ρ* = 0.75	57.3% (47–66.3)
	*ρ* = 0.9	52.9% (43.7–62.5)
Constrained randomisation	*ϕ* = 0.5	79.0% (72–80.5)
	*ϕ* = 0.75	79.8% (78.8–80.2)
	*ϕ* = 0.9	79.9% (79.3–80.2)
BAR		62.6% (54-70.3)

Abbreviation: BAR, Bayesian adaptive randomisation.

**Table 2:** Average proportion of patients on the best treatment available to them

		RFAC			Hierarchy			CR			
Scenario	ER	*θ* = 0.2	*θ* = 0.25	*θ* = 0.3	*ρ* = 0.5	*ρ* = 0.75	*ρ* = 0.9	*ϕ* = 0.5	*ϕ* = 0.75	*ϕ* = 0.9	BAR
Scenario 2	11.5	16.0	15.0	14.0	10.4	12.7	14.0	14.6	14.5	14.3	9.3
Scenario 3	2.1	2.5	2.3	2.2	1.9	2.7	3.3	2.2	2.3	2.4	3.2
Scenario 4	17.5	13.1	14.0	14.8	18.5	16.8	15.6	14.3	14.6	14.8	90.6
Scenario 8	9.3	13.5	12.7	11.8	8.6	9.9	10.7	12.4	12.1	11.9	9.4
Scenario 9	12.9	8.8	9.6	10.4	13.6	12.5	11.7	9.9	10.2	10.4	15.3
Scenario 10	15.4	11.6	12.3	12.9	16.8	14.7	13.9	12.9	13.3	13.5	52.3
Scenario 11	17.0	21.5	20.1	18.8	17.3	23.0	26.4	19.8	20.0	20.0	14.7
Scenario 12	17.0	21.5	20.1	18.8	17.3	23.0	26.4	19.8	20.0	20.0	9.4

*Note:* We exclude scenario 1 as there is no ‘best’ treatment in this case. All treatments induce equal probability of response as control. Abbreviations: BAR, Bayesian adaptive randomisation; CR, constrained randomisation; ER, equal randomisation; RFAC, randomisation with fixed allocation probability to control.

**Table 3:** Comparison of the statistical power for each of the simulation scenarios

Scenario	Treatment allocation approach	Recommend T1 in B1+	Recommend T1 in B2+	Recommend T2 in B1+
Scenario 1: All Tx have same effect as control	ER^a^	5.24%	5.35%	5.22%
	RFAC^a^	4.98%	4.95%	5.27%
	Hierarchy^a^	5.07%	5.61%	1.51%
	CR^a^	4.98%	4.98%	5.41%
	BAR^a^	4.98%	4.22%	4.34%
Scenario 2: T1 works in B1+ only	ER	79.31%	3.95%	5.22%
	RFAC	78.0%	4.10%	5.27%
	Hierarchy	82.57%	5.21%	1.51%
	CR	72.78%	4.15%	5.41%
	BAR	82.74%	5.18%	4.59%
Scenario 3: T1 works in B2+ only	ER	5.24%	33.58%	5.22%
	RFAC	4.92%	32.13%	5.27%
	Hierarchy	5.12%	49.61%	1.51%
	CR	4.66%	33.21%	5.41%
	BAR	5.05%	41.09%	5.38%
Scenario 4: T1 has detrimental effect in B1+	ER	0.05%	3.78%	5.22%
	RFAC	0.02%	4.38%	5.27%
	Hierarchy	0.01%	4.40%	1.51%
	CR	0.01%	4.56%	3.79%
	BAR	0.00%	4.07%	4.50%
Scenario 8: T1 benefits B1+ and harm in B2+	ER	78.96%	0.16%	5.22%
	RFAC	78.17%	0.27%	5.27%
	Hierarchy	81.94%	0.09%	1.51%
	CR	72.65%	0.17%	5.41%
	BAR	81.44%	0.002%	3.86%
Scenario 9: T1 benefits B2+ and harm in B1+	ER	0.02%	24.15%	5.22%
	RFAC	0.02%	26.02%	5.27%
	Hierarchy	0.01%	34.74%	1.51%
	CR	0.02%	26.43%	5.41%
	BAR	0.00%	40.0%	3.43%
Scenario 10: T1 harms B1+, T2 provides benefit in B1	ER	0.05%	3.78%	33.1%
	RFAC	0.02%	4.38%	32.34%
	Hierarchy	0.01%	4.40%	3.90%
	CR	0.01%	4.74%	39.77%
	BAR	0.00%	5.69%	27.04%
Scenario 11: T1 provides some benefit for all	ER	11.89%	8.06%	5.22%
	RFAC	10.74%	7.71%	5.27%
	Hierarchy	11.80%	9.76%	1.51%
	CR	10.25%	7.73%	5.41%
	BAR	10.52%	4.11%	8.48%
Scenario 12: Same as 11, works for B1+	ER	89.97%	5.85%	5.22%
	RFAC	89.30%	6.10%	5.27%
	Hierarchy	92.32%	8.65%	1.51%
	CR	85.20%	6.58%	5.41%
	BAR	92.24%	4.38%	6.34%

*Note:* RFAC, Hierarchy and CR evaluated at *θ* = 0.3; *ρ* = 0.9; *ϕ* = 0.75 respectively (as defined in section 3.2). Simulations using different values of *⊖*, *ρ* and *ϕ* have been done but not presented here. Abbreviations: BAR, Bayesian adaptive randomisation; CR, constrained randomisation; ER, equal randomisation; RFAC, randomisation with fixed allocation probability to control.

a Type I error rate.

## Data Availability

Data sharing is not applicable to this article as no new data were created or analyzed in this study.
