# Supplementary material for: Treatment allocation strategies for umbrella trials in the presence of multiple biomarkers: A comparison of methods
Source: Pharm Stat. Author manuscript; Available in PMC 2022 Apr 11. (PMC7612600; doi:10.1002/pst.2119)
Supplement: Supplementary File [file EMS144016-supplement-Supplementary_File.docx]

# Supplementary Material

**Bayesian adaptive randomisation procedure**

The aim of implementing this BAR procedure is specifically to inform allocation of patients who test positive for multiple biomarkers using accumulating trial data. As such, the BAR procedure is not used to guide allocation of patients who test positive for one or none of the biomarkers of interest. We assume a recruitment rate of 8 patients per month over a period of approximately 3 years.

A total of $J-1$ interim analyses are pre-planned over the trial duration for the BAR procedure. Because no adaptation is needed for the other four alternative treatment allocation approaches, we do not have any interim analysis. The interim analyses are equally spaced, each taking place after $n_{j}$ patients have been recruited. A 6-month delay between patient recruitment and outcome assessment is implemented for the simulation study. At the first stage, all patients are equally randomised between the control and experimental treatments for which they are eligible. At the $jth$ interim analysis, the allocation probabilities to treatment arms are only updated to guide the allocation of patients who test positive for multiple biomarkers.

A Bayesian version of the logistic regression model as specified in the main paper is fitted to model the probability of treatment response. The model is fitted using the *R2jags* and *rjags* packages in R, using 10,000 iterations with 2,500 discarded as burn-in.

For all of the parameters of the Bayesian model ($\alpha$,$\beta$and$\gamma$), we specify uninformative uniform prior distributions (-10, 10). For each of the biomarker profiles, we obtain (from Equation 1) the posterior probabilities that each experimental treatment is better than control. More generally, for a biomarker profile given by the vector $x_{i}=(x_{i1},\ldots,x_{ik})$, where $x_{ik}$is either 0 or 1, the posterior probability for a given experimental treatment, say k, being superior to control is given by

$$\mathbb{P}\left( \beta_{k}+\sum_{l=1}^{K} x_{il}\delta_{lk}>0 | trial data \right)$$

The allocation probabilities are updated using Equation 1 via an approach that has previously been described elsewhere; see Wason et al^14^. After all patients have been assessed, a classical logistic regression is used for the final analysis.

**Simulation scenarios**

For each simulation scenarios, we vary the parameters of the model (equation 1) as described in Table S1 below. For each scenario, 10,000 simulation replicates were used.

Table S1: Description of simulation scenarios

| **Scenario number** | **Scenario description** |
| --- | --- |
| Scenario1 – All Tx have same effect as control | All parameters are set to 0, $\alpha$is set to -0.85. |
| Scenario2 – T1 works in B1+ only | As scenario 1, except $\delta_{11}=1.32.$ |
| Scenario3 – T1 works in B2+ only | As scenario 1, except $\delta_{12}=1.32$ |
| Scenario4 – T1 has detrimental effect in B1+ | As scenario 1, except $\delta_{11}=-1.32.$ |
| Scenario5 – T1 has detrimental effect in B2+ | As scenario 1, except $\delta_{12}=-1.32.$ |
| scenario6 – T1 works in B1+ & B2+ only | As scenario 1, except $\delta_{11}=1.32,\delta_{12}=1.32$ |
| Scenario7 – T1 harms B1+ & B2+ | As scenario 1, except $\delta_{11}=-1.32,\delta_{12}=-1.32$ |
| Scenario8 – T1 benefits B1+ and harm in B2+ | As scenario 1, except $\delta_{11}=1.32,\delta_{12}=-1.32.$ |
| Scenario9 – T1 benefits B2+ and harm in B1+ | As scenario 1, except $\delta_{11}=-1.32,\delta_{12}=1.32.$ |
| Scenario10 – T1 harms B1+, T2 provides benefit in B1 | As scenario 1, except $\delta_{11}=-1.32,\delta_{21}=1.32.$ |
| Scenario11 – T1 provides some benefit for all | As scenario 1, except $\beta_{1}=0.25.$ |
| Scenario12 – Same as 11, works for B1+ | As scenario 11, except $\beta_{1}=0.25, \delta_{11}=1.32.$ |

**Best treatment in each simulation scenario**

We define the best treatment in each scenario as that which induces the highest probability of a favourable response given the patients biomarker profile. In scenario1, all treatments have the same probability of response as control, hence no treatment is considered superior. In scenario 2, T1 is the best treatment for patients of biomarker profiles P9-P14. When a treatment offers an unanticipated detrimental effect (scenarios 4, 9 and 10), all other treatments except T_1_ are considered ‘best’.

Figure S1: Best treatment in each simulation scenario





Table S2: Summary of expected biomarker profiles in the simulated datasets

| **Biomarker subgroup** | **Number of patients** | **Proportion (%)** |
| --- | --- | --- |
| No biomarker | 110 | 27.5 |
| B1 | 47 | 11.75 |
| B2 | 37 | 9.25 |
| B3 | 47 | 11.75 |
| B4 | 37 | 9.25 |
| B1, B2 | 16 | 4 |
| B1, B3 | 20 | 5 |
| B1, B4 | 16 | 4 |
| B1, B2, B4 | 5 | 1.25 |
| B1, B3, B4 | 7 | 1.75 |
| B1, B2, B3 | 7 | 1.75 |
| B1, B2, B3, B4 | 2 | 0.5 |
| B2, B4 | 12 | 3 |
| B2, B3 | 16 | 4 |
| B3, B4 | 16 | 4 |
| B2, B3, B4 | 5 | 1.25 |
| **Multiple biomarkers** | 122 | 30.5 |
| **Total** | 400 | 100 |
| *We assume the occurrence of biomarkers is independent | |  |

# Supplementary results

Table S3: Average number of patients allocated to experimental treatments and the control.

|  | |  | **T0*** | **T1** | **T2** | **T3** | **T4** |
| --- | --- | --- | --- | --- | --- | --- | --- |
| Equal Randomisation | |  | 144.4 | 68.1 | 59.7 | 68.1 | 59.8 |
| Randomisation with fixed allocation probability to control | θ = 0.2 | | 80.0 | 86.2 | 73.9 | 85.9 | 74.0 |
|  | θ = 0.25 | | 100.0 | 80.7 | 69.4 | 80.36 | 69.4 |
|  | θ = 0.3 | | 119.9 | 75.3 | 64.7 | 75.2 | 64.8 |
| Hierarchy | ρ = 0.5 | | 141.9 | 69.4 | 56.3 | 69.0 | 63.4 |
|  | ρ = 0.75 | | 170.8 | 92.5 | 45.6 | 50.1 | 40.9 |
|  | ρ = 0.9 | | 188.2 | 106.3 | 39.1 | 39.0 | 27.4 |
| Constrained randomisation | ϕ = 0.5 | | 84.1 | 79.4 | 78.5 | 79.4 | 78.5 |
|  | ϕ = 0.75 | | 80.7 | 79.9 | 79.7 | 79.9 | 79.7 |
|  | ϕ = 0.9 | | 80.4 | 80.0 | 79.8 | 80.0 | 79.8 |
| BAR | |  | 149.5 | 65.5 | 61.0 | 65.8 | 58.2 |

*T0 is control treatment; T1-T4 are biomarker-linked treatments;

Table S4: Treatment response across different treatment allocation approaches

|  |  | **T0*** | **T1** | **T2** | **T3** | **T4** |
| --- | --- | --- | --- | --- | --- | --- |
| Equal Randomisation |  | 36 (15-61) | 8.7-35 (1-58) | 14.9 (2-30) | 17 (14-32) | 15 (2-31) |
| Randomisation with fixed probability of allocation to control | θ = 0.2 | 20 (5-37) | 10.1-46.1 (1-73) | 18.4 (5-44) | 21.5 (18-41) | 18.6 (5-35) |
|  | θ = 0.25 | 25 (10-44) | 9.4-43.1 (1-68) | 17.3 (3-36) | 20.1 (6-40) | 17.4 (4-33) |
|  | θ = 0.3 | 30 (12-52) | 8.8-40.3 (0-65) | 16.1 (4-36) | 18.8 (5-36) | 16.2 (4-33) |
| Hierarchy | ρ = 0.5 | 35.4 (16-61) | 9.9-34.1(1-60) | 14.1 (2-33) | 17.2 (4-35) | 15.9 (3-32) |
|  | ρ = 0.75 | 42.7 (22-70) | 13.9-43.9 (3-67) | 11.4 (1-26) | 12.5 (2-27) | 10.2 (1-26) |
|  | ρ = 0.9 | 47 (24-77) | 17-49.7 (4-82) | 9.8 (0-23) | 9.7 (0-22) | 6.8 (0-19) |
| Constrained randomisation | ϕ = 0.5 | 21 (7-38) | 9.4-42.3 (1-59) | 19.7 (5-42) | 19.8 (6-36) | 19.7 (6-35) |
|  | ϕ = 0.75 | 20.1 (5-35) | 9.6-42.3 (1-60) | 19.9 (5- 40) | 20 (7-35) | 20 (7-36) |
|  | ϕ = 0.9 | 20.0 (7-35) | 9.7-42.1 (0-58) | 19.9 (6-40) | 20.0 (6-34) | 20 (7-37) |
| BAR |  | 37.3 (18-57) | 16.1 (4-30) | 15.1 (4-32) | 16.5 (3-31) | 14.5 (3-29) |

*T0 is control; T1-T4 are biomarker-linked treatments;

Table S5: Statistical power of the Randomisation with fixed probability of allocation to control as we vary $\theta$

|  | $\theta=0.5$ | | | $\theta=0.75$ | | | $\theta=0.9$ | | |
| --- | --- | --- | --- | --- | --- | --- | --- | --- | --- |
|  | Recommend T1 in B1+ | Recommend T1 in B2+ | Recommend T2 in B1+ | Recommend T1 in B1+ | Recommend T1 in B2+ | Recommend T2 in B1+ | Recommend T1 in B1+ | Recommend T1 in B2+ | Recommend T2 in B1+ |
| **Scenario1** – All Tx have same effect as control | 4.67% | 4.66% | 5.72% | 5.02% | 5.10% | 5.36% | 4.98% | 4.95% | 5.27% |
| **Scenario2** – T1 works in B1+ only | 68.63% | 4.59% | 5.72% | 74.36% | 4.27% | 5.36% | 78.00% | 4.10% | 5.27% |
| **Scenario3** – T1 works in B2+ only | 4.55% | 32.99% | 5.72% | 4.96% | 33.33% | 5.36% | 4.92% | 32.13% | 5.27% |
| **Scenario4** – T1 has detrimental effect in B1+ | 0.06% | 4.93% | 5.72% | 0.04% | 4.51% | 5.36% | 0.02% | 4.38% | 5.27% |
| **Scenario5** – T1 has detrimental effect in B2+ | 4.71% | 0.34% | 5.72% | 5.10% | 0.38% | 5.36% | 5.03% | 0.39% | 5.27% |
| **scenario6** – T1 works in B1+ & B2+ only | 68.55% | 18.36% | 5.72% | 74.19% | 17.27% | 5.36% | 77.68% | 15.67% | 5.27% |
| **Scenario7** – T1 harms B1+ & B2+ | 0.05% | 0.76% | 5.72% | 0.03% | 0.84% | 5.36% | 0.02% | 0.77% | 5.27% |
| **Scenario8** – T1 benefits B1+ and harm in B2+ | 68.57% | 0.29% | 5.72% | 74.61% | 0.27% | 5.36% | 78.17% | 0.27% | 5.27% |
| **Scenario9** – T1 benefits B2+ and harm in B1+ | 0.06% | 26.67% | 5.72% | 0.05% | 26.91% | 5.36% | 0.02% | 26.02% | 5.27% |
| **Scenario10** – T1 harms B1+, T2 provides benefit in B1 | 0.06% | 4.93% | 34.51% | 0.04% | 4.51% | 33.26% | 0.02% | 4.38% | 32.34% |
| **Scenario11** – T1 provides some benefit for all | 9.52% | 7.81% | 5.72% | 10.41% | 7.91% | 5.36% | 10.74% | 7.71% | 5.27% |
| **Scenario12** – Same as 11, works for B1+ | 81.83% | 6.87% | 5.72% | 86.58% | 6.46% | 5.36% | 89.30% | 6.10% | 5.27% |

Table S6: Statistical power of the Hierarchy approach as we vary $\rho$

|  | $\rho=0.5$ | | | $\rho=0.75$ | | | $\rho=0.9$ | | |
| --- | --- | --- | --- | --- | --- | --- | --- | --- | --- |
|  | Recommend T1 in B1+ | Recommend T1 in B2+ | Recommend T2 in B1+ | Recommend T1 in B1+ | Recommend T1 in B2+ | Recommend T2 in B1+ | Recommend T1 in B1+ | Recommend T1 in B2+ | Recommend T2 in B1+ |
| **Scenario1** – All Tx have same effect as control | 5.11% | 5.17% | 5.70% | 5.27% | 5.70% | 4.38% | 5.07% | 5.61% | 1.51% |
| **Scenario2** – T1 works in B1+ only | 78.03% | 3.87% | 5.70% | 80.86% | 4.95% | 4.38% | 82.57% | 5.21% | 1.51% |
| **Scenario3** – T1 works in B2+ only | 5.08% | 30.13% | 5.70% | 5.14% | 43.79% | 4.38% | 5.12% | 49.61% | 1.51% |
| **Scenario4** – T1 has detrimental effect in B1+ | 0.02% | 3.70% | 5.70% | 0.02% | 4.10% | 4.38% | 0.01% | 4.40% | 1.51% |
| **Scenario5** – T1 has detrimental effect in B2+ | 5.15% | 0.46% | 5.70% | 5.33% | 0.17% | 4.38% | 5.23% | 0.23% | 1.51% |
| **scenario6** – T1 works in B1+ & B2+ only | 77.50% | 11.90% | 5.70% | 79.94% | 26.08% | 4.38% | 81.81% | 34.28% | 1.51% |
| **Scenario7** – T1 harms B1+ & B2+ | 0.02% | 0.51% | 5.70% | 0.03% | 0.57% | 4.38% | 0.01% | 0.41% | 1.51% |
| **Scenario8** – T1 benefits B1+ and harm in B2+ | 77.90% | 0.19% | 5.70% | 80.12% | 0.14% | 4.38% | 81.94% | 0.09% | 1.51% |
| **Scenario9** – T1 benefits B2+ and harm in B1+ | 0.03% | 21.35% | 5.70% | 0.03% | 30.32% | 4.38% | 0.01% | 34.74% | 1.51% |
| **Scenario10** – T1 harms B1+, T2 provides benefit in B1 | 0.02% | 3.68% | 36.27% | 0.02% | 4.10% | 20.10% | 0.01% | 4.40% | 3.90% |
| **Scenario11** – T1 provides some benefit for all | 11.14% | 7.83% | 5.70% | 11.46% | 9.39% | 4.38% | 11.80% | 9.76% | 1.51% |
| **Scenario12** – Same as 11, works for B1+ | 88.98% | 5.44% | 5.70% | 91.10% | 7.64% | 4.38% | 92.32% | 8.65% | 1.51% |

Table S7: Statistical power of the constrained randomisation approach as we vary $\phi$

|  | $\phi=0.5$ | | | $\phi=0.75$ | | | $\phi=0.9$ | | |
| --- | --- | --- | --- | --- | --- | --- | --- | --- | --- |
|  | Recommend T1 in B1+ | Recommend T1 in B2+ | Recommend T2 in B1+ | Recommend T1 in B1+ | Recommend T1 in B2+ | Recommend T2 in B1+ | Recommend T1 in B1+ | Recommend T1 in B2+ | Recommend T2 in B1+ |
| **Scenario1** – All Tx have same effect as control | 4.97% | 4.97% | 5.87% | 4.98% | 4.98% | 5.41% | 4.70% | 5.00% | 5.68% |
| **Scenario2** – T1 works in B1+ only | 72.64% | 4.01% | 5.87% | 72.78% | 4.15% | 5.41% | 73.11% | 4.10% | 5.68% |
| **Scenario3** – T1 works in B2+ only | 4.94% | 31.96% | 5.87% | 4.66% | 33.21% | 5.41% | 4.64% | 33.30% | 5.68% |
| **Scenario4** – T1 has detrimental effect in B1+ | 0.03% | 4.57% | 5.87% | 0.01% | 4.74% | 5.41% | 0.04% | 4.70% | 5.68% |
| **Scenario5** – T1 has detrimental effect in B2+ | 5.02% | 0.42% | 5.87% | 4.94% | 0.34% | 5.41% | 4.84% | 0.40% | 5.68% |
| **scenario6** – T1 works in B1+ & B2+ only | 72.33% | 15.70% | 5.87% | 72.18% | 17.18% | 5.41% | 72.79% | 17.30% | 5.68% |
| **Scenario7** – T1 harms B1+ & B2+ | 0.03% | 0.72% | 5.87% | 0.00% | 0.85% | 5.41% | 0.05% | 0.70% | 5.68% |
| **Scenario8** – T1 benefits B1+ and harm in B2+ | 72.44% | 0.22% | 5.87% | 72.65% | 0.17% | 5.41% | 73.20% | 0.20% | 5.68% |
| **Scenario9** – T1 benefits B2+ and harm in B1+ | 0.02% | 25.84% | 5.87% | 0.02% | 26.43% | 5.41% | 0.04% | 26.70% | 5.68% |
| **Scenario10** – T1 harms B1+, T2 provides benefit in B1 | 0.03% | 4.57% | 38.09% | 0.01% | 4.74% | 39.77% | 0.04% | 4.70% | 39.93% |
| **Scenario11** – T1 provides some benefit for all | 10.54% | 7.69% | 5.87% | 10.25% | 7.73% | 5.41% | 10.38% | 7.80% | 5.68% |
| **Scenario12** – Same as 11, works for B1+ | 85.13% | 6.03% | 5.87% | 85.20% | 6.58% | 5.41% | 85.35% | 6.40% | 5.68% |

Table 4: Comparison of the bias for each of the simulation scenarios

| Scenario | Treatment  allocation approach | Recommend T1 in B1+ | Recommend T1 in B2+ | Recommend T2 in B1+ |
| --- | --- | --- | --- | --- |
| Scenario 1: All Tx have same effect as control | ER | 0.057 | -1.512 | -1.614 |
|  | RFAC | 0.16 | -1.369 | -1.343 |
|  | Hierarchy | -0.004 | -2.157 | -0.91 |
|  | CR | 0.119 | -1.176 | -0.707 |
|  | BAR | 0.028 | -1.279 | -0.788 |
| Scenario 2: T1 works in B1+ only | ER | 0.157 | 0.244 | -1.596 |
|  | RFAC | 0.219 | 0.223 | -1.33 |
|  | Hierarchy | 0.109 | 0.332 | -0.901 |
|  | CR | 0.185 | 0.229 | -0.701 |
|  | BAR | 0.171 | -0.104 | -1.02 |
| Scenario 3: T1 works in B2+ only | ER | 0.043 | 0.596 | -1.605 |
|  | RFAC | 0.155 | 0.439 | -1.336 |
|  | Hierarchy | -0.007 | 1.061 | -0.903 |
|  | CR | 0.112 | 0.431 | -0.704 |
|  | BAR | 0.056 | -1.699 | 1.104 |
| Scenario 4: T1 has detrimental effect in B1+ | ER | -1.836 | -5.675 | -1.685 |
|  | RFAC | -0.65 | -6.016 | -1.404 |
|  | Hierarchy | -2.244 | -6.156 | -0.949 |
|  | CR | -0.837 | -5.656 | -0.737 |
|  | BAR | 0.057 | -1.512 | -1.614 |
| Scenario 8: T1 benefits B1+ and harm in B2+ | ER | 0.162 | -1.778 | -1.602 |
|  | RFAC | 0.221 | -1.558 | -1.335 |
|  | Hierarchy | 0.113 | -2.77 | -0.905 |
|  | CR | 0.188 | -1.348 | -0.702 |
|  | BAR | 0.139 | -0.348 | -6.192 |
| Scenario 9: T1 benefits B2+ and harm in B1+ | ER | -1.603 | -0.13 | -1.656 |
|  | RFAC | -0.553 | -0.771 | -1.378 |
|  | Hierarchy | -2.023 | -0.351 | -0.936 |
|  | CR | -0.692 | -0.504 | -0.722 |
|  | BAR | -1.534 | -3.065 | 0.709 |
| Scenario 10: T1 harms B1+, T2 provides benefit in B1 | ER | -1.832 | -5.648 | 0.831 |
|  | RFAC | -0.649 | -5.992 | 0.605 |
|  | Hierarchy | -2.237 | -6.122 | 0.501 |
|  | CR | -0.836 | -5.633 | 0.32 |
|  | BAR | -1.535 | 0.899 | -0.586 |
| Scenario 11: T1 provides some benefit for all | ER | 0.034 | -1 | -1.605 |
|  | RFAC | 0.091 | -0.862 | -1.335 |
|  | Hierarchy | 0.002 | -1.509 | -0.905 |
|  | CR | 0.089 | -0.699 | -0.704 |
|  | BAR | 0.044 | -0.8 | -0.694 |
| Scenario 12: Same as 11, works for B1+ | ER | 0.12 | 0.514 | -1.597 |
|  | RFAC | 0.149 | 0.462 | -1.33 |
|  | Hierarchy | 0.096 | 0.722 | -0.902 |
|  | CR | 0.153 | 0.395 | -0.702 |
|  | BAR | 0.115 | -0.111 | -1.032 |
| ER = Equal randomisation; RFAC = Randomisation with fixed allocation probability to control; CR = Constrained randomisation; BAR = Bayesian adaptive randomisation.  RFAC, Hierarchy and CR evaluated at $\boldsymbol{\theta=0.3}$; $\boldsymbol{\rho= 0.9}$; $\boldsymbol{\phi= 0.75}$ respectively (as defined in section 3.2). Simulations using different values of ϴ, ρ and ϕ have been done but not presented here. | | | | |
